# Adsorptive removal of phosphate from water with biochar from acacia tree modified with iron and magnesium oxides

**DOI:** 10.1038/s41598-024-66965-3

**Published:** 2024-07-29

**Authors:** Yehia Manawi, Rashad Al-Gaashani, Simjo Simson, Yongfeng Tong, Jenny Lawler, Viktor Kochkodan

**Affiliations:** 1 Qatar Environment and Energy Research Institute, Hamad Bin Khalifa University, Qatar Foundation, PO Box 34110, Doha, Qatar; 2 HBKU Core Labs, Hamad Bin Khalifa University, Qatar Foundation, Doha, Qatar

**Keywords:** Materials science, Pollution remediation

## Abstract

A novel biochar (BC) from *Acacia* *tortilis* trees pruning waste was synthesized and tested for the removal of phosphate from aqueous solutions. The BC was prepared by calcination at 600 °C and doped with Fe_3_O_4_ and MgO by hydrothermal process. The presence of iron and magnesium ions in the modified BC was confirmed by EDS analysis and X-ray diffraction (XRD) methods. Both unmodified and doped BCs were tested for phosphate removal from synthetic 1–500 ppm aqueous solutions. While the unmodified BC did not show any significant removal of phosphate from aqueous solutions, the modified BC almost completely removed phosphate from water. The enhancement in removal efficiency is due to an increase in the overall surface charge and surface area of BC as a result of doping with Fe_3_O_4_ and MgO salts. The average porosity and BET surface area corresponding to the plain BC increased by more than 20% from 322 to 394 m^2^/g after modification by impregnation with iron oxide and magnesium oxide. The modificaiton of BC with Fe_3_O_4_ and MgO nanoparticles was observed to increase the point of zero electric charge (PZC) from pH 3.4 (corresponding to plain BC) to pH 5.3 (corresponding to modified BC). The adsorption process was very fast and a phosphate removal value of 82.5% was reached only after 30 min of adsorption, while the removal efficiency after 4 h of adsorption was 97.5%. The rapid removal efficiency in short contact time is attributed to the high surface area of BC and strong bonding between the modified BC surface and PO_4_^3−^ ions. The highest adsorption capacity was observed to correspond to 98.5 mg/g which was achieved at PO_4_^3−^ concentration of 500 ppm and pH 8.5. Moreover, after fitting the adsorption data onto four of the most widely used adsorption isotherm models, the adsorption of PO_4_^3−^ onto BC can be better described by the Langmuir isotherm model.

## Introduction

Pure phosphorus element (P) cannot be found by itself in nature as a result of its high reactivity hence it is found in different forms such as phosphates in rocks and in living organisms^1^. Both phosphorus and phosphates are critical for the growth and repair of all living cells as well as building our DNA. The typical content of organic and inorganic phosphate in a 70 kg person is about 0.7 kg which accounts for around 1.1% of the body weight^2^. They are essential for plants wellbeing and that’s why phosphates are added to the soil as a source of nutrient^3^. It was estimated that the production of 70% of the global phosphate comes from natural reserves which are going to deplete within 100 years and the difference between the demand and production of phosphate in 2070 will be greater than the existing annual production rate^4^. Moreover, more than 80% of the mined phosphate is used as a fertilizer for the soil^5^. A substantial fraction of the phosphate used in fertilizers winds up in natural water bodies or sewage water streams through agricultural runoff causing eutrophication or depletion of dissolved oxygen in water due to extreme uptake by algae feeding on phosphate^6^. The estimated global amounts of phosphorous released from wastewater plants is 3 million metric tons per year^7^. About 80% of the phosphate in lakes and rivers comes from agricultural runoff^8^.

The removal of phosphate from wastewater can be performed by either biological treatment or chemical precipitation; however, chemical precipitation is related to high cost of coagulants and generation of secondary wastes^9^. Biological treatment involves incorporating phosphorus into the cell biomass of some microorganisms and has been used for a long time due to their cost-effectiveness at large scale compared to chemical precipitation. However, biological treatment does not remove phosphorus from wastewater treatment plants effluents completely. A survey conducted by Minnesota pollution control agency on 59 wastewater treatment plants using activated sludge to remove phosphorus reported the average removal efficiency of phosphorus to be 47%^10^. Another survey conducted to determine the phosphate level in the rivers of Switzerland found out the phosphate level to range between 0.11 and 0.37 mg/L^11^ which was higher than the level reported by the Environmental Protection Agency (EPA) of 0.035 mg/L and can cause harm to other plants and animals^12^. According to the EPA, more than one-third of the rivers in the USA have phosphate levels higher than 0.035 mg/L and 25% of the rivers in the USA were reported show increasing phosphate levels^12^. Hence, the recycling of the phosphate from wastewater will not only have economic benefits but will also have environmental benefits. The aim of the present work is to recycle phosphate by removing it from the wastewater effluents using novel modified biochar (BC).

The removal of phosphate from water using BC prepared from various sources have been reported in literature^13–17^; however, there are some drawbacks which need to be addressed such as rather low surface area, adsorption capacity and removal rate of the sorbents. For instance, the BC adsorbent prepared by Jung et al.^17^ from peanut shells to remove phosphate from water was found to show low phosphate removal rate (61%) and rather low adsorption capacities (6.7 mg/g). Likewise, BC prepared from *Broussonetia* *papyrifera* leaves was tested for the removal of phosphate from water and showed a low BET surface area (9.5 m^2^/g) and adsorption capacity (< 8 mg/g) at phosphate solution concentration of 50 mg/L^18^. The phosphate adsorbent prepared by Lee et al.^19^ by calcination of egg shells at various calcination temperatures (ranging between 100 and 900 °C) under nitrogen showed that the best adsorbent corresponded to a calcination temperature of 800 °C. This adsorbent was tested with real lake water and showed the phosphate adsorption capacity of 11.5 mg/g after 158 h adsorption time.

The BC modification by various additives such as magnesium, aluminum, calcium and lanthanum for the removal of phosphate from water was reported in literature to increase the BET surface area as well as porosity^13–17,20^. For instance, the modification of BC by magnesium and aluminum was reported to increase the surface area by 18 and 26%, respectively^14^ whereas the modification of BC with calcium enhanced the BET surface area from 0.19 to 79.9 m^2^/g^17^. The enhancement in the surface area of an adsorbent has the effect of increasing the adsorption rate due to the rise in the contact between adsorbent and sorbent^21^. It was shown that because both BC and phosphate are negatively charged in water, the phosphate adsorption onto BC is controlled by electrostatic interactions^22^. Moreover, BC impregnated with metals was reported to increase the adsorption performance due to shifting the point of zero electric charge to a higher pH^23^. For instance, the pH_PZC_ of plain BC and BC modified with MgO (BC/MgO) for the removal of phosphate from water were reported at 8.2 and 10.8, respectively^24^. The increase in the pH_PZC_ was reported to effectively increase the electrostatic attractive forces between PO_4_^3−^ ions and BC/MgO keeping in mind that the pH values of all the studied adsorption solutions were below pH_PZC_ of BC/MgO of 10.8. Furthermore, Kang et al.^25^ tested the phosphate adsorption onto (a) food waste loaded with iron (FW-Fe) as well as (b) BC prepared from food waste, which was modified with iron (FWB-Fe). The loading of iron onto both adsorbents was performed using iron chloride precursor. The adsorption capacity of FWB-Fe was reported to be higher than that of FW-Fe by more than 76% due to greater specific area (by more than 50%), well-developed pores as well as uniform distribution of iron onto the surface of FWB-Fe. Despite that, the optimum BC modified with iron showed a maximum phosphate adsorption capacity of 31.8 mg/g which was quite low.

In addition, BC modification with metals oxide such as Fe_2_O_3_ and MgO was also reported in literature to enhance adsorption of other contaminants from water. For instance, the BC modified with iron oxide by Peng et al.^26^ showed smaller carbon particle size when compared with plain BC which was attributed to the presence of iron during carbonization of biomass. This was supported by BET surface area of pure BC and BC modified with iron oxide which showed BET surface areas of 338 and 527 m^2^/g, respectively. The experimental tests showed that the modified BC outperformed plain BC towards rhodamine B with adsorption capacities of 289.6 and 179.7 mg/g, respectively. Likewise, BC modification with Fe_2_O_3_ was reported by Xu et al.^27^ to increase the BET surface area from 32.9 to 284.4 m^2^/g and reduce average pore size from 1819 to 213 nm. This was confirmed by SEM images which showed Fe_2_O_3_ taking the shape of snow nanorods with average lengths ranging between 2 and 50 nm. The adsorption capacity of plain BC towards quinoline in water was reported to increase from 20.8 to 33.3 mg/g after the modification with Fe_2_O_3_.

Similarly, the BC modified with MgO by Shi et al.^28^ for the removal of lead and cadmium form water was reported to exhibit greater surface area as well as widely available adsorption sites due to the formation of pore structure within plain BC after the modification with MgO. The MgO-modified BC was reported to show adsorption capacities of 829 and 515 mg/g towards cadmium and lead, respectively with high removal efficiency of 99.9%.

Owing to its outstanding characteristics such as resistance to diseases and severe climatic conditions^29,30^, *Acacia* *tortilis* trees are one of the prominent species that are found abundantly in different parts of the world and dominate eastern, northern and southern Africa all the way to the Arabian Peninsula^31,32^. These trees are the key source for providing food for livestock and wood fuel in addition to numerous other applications. Not only the trees are used for the production but also for the protection; such trees are used as windbreak to achieve sand stabilization as well as reducing the ambient temperature through the cooling effect of both shade and evapotranspiration^31^. In Qatar, *Acacia* *tortilis* tree which is locally known as Samar, is considered one of the national trees of Qatar. These trees are found widely in parks, streets, landscapes, biosphere reserves, etc. They are pruned regularly and generate huge amounts of waste biomass. It was reported that the amount of *Acacia* *mangium* bark wastes disposed by a medium-sized tanning-extract plant is in China was estimated at about 1000 ton annually^33^. This biomass waste was also reported to naturally contain numerous elements such as: Mg, Mn, Mo, Zn, Co, Cu, Fe and Se, etc.^29^ which enhance the adsorbent’s characteristic sorption capacity.

While the use of acacia tree biomass to prepare BC was reported in literature^33–36^, to the best of our knowledge, the use of *Acacia* *tortilis* biomass to develop modified BC by Fe_3_O_4_ and MgO for the removal of phosphate from wastewater was not investigated yet. The utilization of this biomass to synthesize the BC could not only help in reusing the biomass wastes but also in recycling the phosphates in wastewater back to the soil. The aim of the present work is to prepare a novel BC from acacia trees modified with Fe_3_O_4_ and MgO for the removal of phosphate from synthetic aqueous solutions.

## Materials and methods

### Materials

Sodium hydroxide (NaOH, 98.0%), iron (III) nitrate nonahydrate (99.99%) and magnesium nitrate hexahydrate (99.99%) were acquired from Sigma Aldrich (Missouri, USA). The preparation of all aqueous solutions in the present work was performed with deionized water (DIW) which has a conductivity of 18.2 MΩ/cm.

### Preparation methods

The PO_4_^3−^ solutions used in the present study were prepared from 1000 mg/L PO_4_^3−^ standard solution (Fisher Scientific, New Hampshire, United States). DIW was used for the dilution of the PO_4_^3−^ standard solutions. The BC used in the present work was prepared from biomass waste after the pruning of acacia trees (*Acacia* *tortilis*). The trunk of the acacia tree was cut into small pieces (2 mm) before taking them to oven for calcination at 600 °C for 4 h. The BC was then ground to fine powder before their modification by the hydrothermal process of Fe_3_O_4_ and MgO at 180 °C for 20 h. The modification was carried out by preparing three BC samples with three different Fe_3_O_4_ and MgO loadings (2, 7 and 15%). Three portions of BC (10 g) were mixed with 100 mL of 2, 7 and 15% iron (III) nitrate nonahydrate and magnesium nitrate hexahydrate (1 M), and NaOH (2 M) solutions before placing them in ultrasonic bath for 30 min. This was then taken to an autoclave in order to be heated for 20 h at 180 °C. The BC-doped Fe_3_O_4_ and MgO was then centrifuged, washed 3 times and calcined at 450 °C for 2 h in air. The optimum Fe_3_O_4_ and MgO loading was determined after conducting adsorption experiments on the three loadings.

### Characterizations

The morphology and structural composition of the BC was analyzed using field emission scanning electron microscope (QUANTA FEG 650, Thermo Fisher Scientific, Massachusetts, USA) which was linked to an X-ray diffraction (XRD) (Bruker D8 Advance X-Ray diffractometer with radiation source of Cu-Kα, Massachusetts, USA) in addition to energy dispersive spectrometer (EDS) (Bruker Xflash 6l60, Karlsruhe, Germany). The surface and porous properties of BC and BC-doped materials were carried out using Brunauer–Emmett–Teller (BET) (ASAP-2020 surface, Micromeritics Instrument Corporation, Georgia, USA). The X-ray photoelectron spectroscopy (XPS) analysis is done using Thermo Fisher Escalab250Xi (Massachusetts, USA). A hemisphere analyzer as well as Al Kα beam that is monochromatic (1486.8 eV) are used, with a entire energy resolution exceeding 0.5 eV. The X-ray is incident at 45°, and a take-off angle of 90° to the plane of sample surface is applied for signal capture. A 100-eV pass energy as well as 1 eV energy step were used for the survey spectra in the present work. Moreover, a 0.1 eV energy step as well as a 50-eV pass energy were selected for high-resolution spectra. The whole measurements are performed in an ultra-high vacuum (10–10 mbar) at room temperature. The calibration of binding energies to the C–C signal (C1s) at 284.8 eV was done. The deconvolution of the spectra was done using a Voigt-type profile (GL (30)) following a appropriate subtraction of Shirley background.

### Batch adsorption experiments

The batch adsorption experiments were carried out as follows. BC was mixed with 100 mL of PO_4_^3−^ solution inside a 250 mL flask. The solutions were then mixed well using Grant OLS Aqua Pro temperature-controlled shaker (Model OLS26, Shepreth, UK). Before samples analysis (when analysis time is due), the filtration of samples through a PTFE membrane syringe filter with (0.22 μm) was performed and the level of PO_4_^3−^ ions in the permeate was then analyzed as will be explained in the following section. The evaluation of the phosphate level in the collected samples was carried out using Thermo Fisher Scientific IC6000 (Massachusetts, USA) and Hach SL1000 (Iowa, USA). Before the analysis, a calibration curve was established by preparing phosphate solutions at various concentrations. The preparation of the calibration solutions was performed by dilution of the 200 mg/L PO_4_^3−^ Dionex seven anion standard II stock calibration standard solution (Thermo Fisher Scientific, California, USA) to generate enough points that will shape the calibration curve.

Equations (1) and (2) were used to determine the removal efficiency of phosphate from water (%) in addition to the adsorption capacity (q_e_, in mg of PO_4_^3−^/g of BC):

1 $$Removal \;Efficiency\; (\% ) = \frac{{(C_{0} - C_{e} )}}{{C_{0} }} \times 100$$

2 $$q_{e} = \frac{{(C_{0} - C_{e} ) \times V}}{m}$$

where, *C*_0_ as well as $C_{e}$ are the first and final phosphate levels in water (mg/L), *m* is the BC mass (g) while *V* is the volume of the phosphate solution (L). The adsorption tests were repeated 3 times. The mean values for all parameters were reported in the present work.

In the present work, the impact of loading of BC was examined by varying the BC dose from 0.5 to 3 g/L. Moreover, the consequence of increasing temperature and shaking speed on the adsorption performance was analyzed by changing the temperature range between 25 and 45 °C and the shaking speed between 100 and 200 rpm. The relationship between acidity and basicity of a solution (pH) on the adsorption performance of BC was analyzed over a range of 2–10. pH was altered by adding HCl as well as NaOH aqueous solutions to adsorption systems containing BC and PO_4_^3−^ solutions. The investigation of adsorption kinetics or impact of time on adsorption performance was investigated after various residence times (1 min to 24 h).

The impact of co-existing ions on phosphate adsorption by BC was also investigated here. Adsorption experiments were conducted under the effect of coexisting ions by carrying out the adsorption tests in a multicomponent mixture of phosphate, chloride and sulfate ions at a 10 mg/L concentration. The complex mixture was made by using 1000 ppm phosphate standard solution (Fisher Scientific, Loughborough, UK), 1000 ppm ion chromatography standard for chloride (Reagecon, Shannon Free Zone, Clare, Ireland) and 1000 ppm sulfate standard for IC (Sigma-Aldrich, Missouri, USA).

Eventually, the regeneration of biochar was investigated by immersion of exhausted BC in basic (0.1 M NaOH) in addition to acidic (0.1 M HCl) media. Next, the BC was filtered out and washed properly with 25 mL of DW. The phosphate content in the DI was determined as has been described above in section “Batch adsorption experiments”. Lastly, the retained BC was oven dried at 80 °C for 16 h. The same adsorption experiments were carried out using the regenerated BC (2 g/L BC in 25 mL of PO_4_^3−^ solution at 10 mg/L concentration).

### Adsorption kinetics

The kinetics of phosphate adsorption process onto BC was investigated by fitting the experimental data into four well known kinetic models which are: pseudo-1st order, 2nd-order, Elovich in addition to intra-particle diffusion. Table 1 shows the equations used to conduct the adsorption kinetic studies.

**Table 1:** Mathematical representation of the four adsorption kinetic models investigated in the present study.

Model	Equation	Linear form	XY plot
Pseudo 1st order	$\frac{{dq_{t} }}{dt} = k_{1} (q_{e} - q_{t} )$	$ln(q_{e} - q_{t} ) = ln(q_{e} )$ − k_1_t	q_t_ vs ln t
Pseudo 2nd order	$\frac{{dq_{t} }}{dt} = k_{2} (q_{e} - q_{t} )^{2}$	$\frac{t}{{q_{t} }} = \frac{1}{{k_{2} q_{e}^{2} }} + \left( {\frac{1}{{q_{e} }}} \right)t$	q_t_ vs ln t
Elovich	$\frac{{dq_{t} }}{dt} = \alpha \;\exp ( - \beta q_{t} )$	$q_{t} = \frac{1}{\beta }ln(\alpha \beta ) + \left( {\frac{1}{\beta }} \right)ln(t)$	q_t_ vs ln t
Intra-particle diffusion	$q_{t} = K_{IPD} \sqrt t + c$	$q_{t} = K_{IPD} \sqrt t + c$	q_t_ vs $\sqrt t$

Where adsorption capacity at any given time (t) as well as equilibrium adsorption capacity are denoted by q_t_ and q_e_, respectively (in mg/g). Moreover, rate constants corresponding to the pseudo 1st and 2nd order models (in 1/min and g/mg/min) are denoted by k_1_ and k_2_, respectively. Moreover, Elovich and intra-particle diffusion model’s parameters (α: initial rate of adsorption (in g/mg) as well as desorption (in mg/g/min) were used. Similarly, intraparticle intercept (in mg/g) as well as intraparticle intercept constant (in mg/g·min) were denoted by K_IPD_ as well as C, respectively^37^.

Equations (3 and 4) were used to determine the half-life time or the time needed for adsorption of half of the PO_4_^3−^ from the solution onto BC^38^:

3 $$t_{1/2} = \frac{ln \;2}{{k_{1} }}$$

4 $$t_{1/2} = \frac{1}{{k_{2} q_{e} }}$$

### Adsorption isotherms

The adsorption isotherms is a term used to describe various adsorption measurements at constant temperature for the sake of plotting the relationship between the adsorbed and non-adsorbed amounts^39^. The shape of the relationship can give an indication of adsorption process. The experimentally measured adsorption capacity was plotted against the concentration of feed PO_4_^3−^ solutions. Adsorption isotherms were studied at a concentration range of 1–500 ppm, shaking speed of 150 rpm and at room temperature. Four of the widely reported models were studied in the present work which are: Langmuir, Sips, Freundlich and Temkin. Table 2 lists the four models employed to fit the adsorption experimental data.

**Table 2:** The four adsorption isotherm models employed in the present study.

Model	Non-linear equation	Parameters
Freundlich	$q_{e} = K_{F} C_{e}^{\frac{1}{n}}$	*K*_*F*_*:* Freundlich constant [in (mg/g)/(dm^3^/mg)^n^] *n:* heterogeneity factor
Langmuir	$q_{e} = \frac{{q_{m} K_{L} C_{e} }}{{1 + K_{L} C_{e} }}$	*K*_*L*_*:* Langmuir constant *q*_*m*_*:* monolayer’s largest adsorption capacity
Sips	q_e_ = $\frac{{q_{max} K_{s} C_{e}^{\frac{1}{n}} }}{{1 + K_{s} C_{e}^{\frac{1}{n}} }}$	*n*: constant of Sips *Ks*: model exponent of Sips
Temkin	$q_{e} = \frac{{RTln\left( {K_{t} C_{e} } \right)}}{b}$	*b*: variation in adsorption energy values parameter (in J/mol) parameter *K*_*t*_: constant of equilibrium binding (in L/g)

The Freundlich model assumes multilayer adsorption on homogenous sites^40^. The model also assumes that the affinities (toward the heterogenous surface) and distribution of adsorption heat are nonuniform^40,41^.

Unlike the Freundlich isotherm model, which assumes multilayer adsorption, the Langmuir model assumes monolayer and homogenous adsorption process. This model was found on the assumption of homogeneity of the adsorbent surface and uniformity of the adsorption energy for all sites^42^. Moreover, the model assumes no migration of adsorbate and no interaction between nearby adsorbate particles^39^.

Equation (5) was employed to estimate the separation factor (*R*_*L*_) that provides an insight about the favorability of adsorption process. *R*_*L*_ value of 0 means that adsorption process is irreversible. Oppositely, favorable adsorption processes were reported to exhibit *R*_*L*_ falling within a range of 0–1. An *R*_*L*_ = 1 denotes linear adsorption process. Conversely, an *R*_*L*_ value > 1 indicates an unfavorable adsorption process^43^.

5 $$R_{L} = \frac{1}{{1 + K_{L} C_{0} }}$$

The Sips adsorption isotherm merges together the Freundlich as well as Langmuir isotherms in order to overcome one of the limitations of the Freundlich equation which is related to the constant increase in the adsorbed quantity along with increase in adsorbate concentration. The Sips model can be used to describe monolayer adsorption over heterogenous surfaces under the condition that there is no interactions between adsorbate molecules^44^. The Temkin model on the other hand assumes multilayer process with some interactions between adsorbate and adsorbent^45^ and that the adsorption heat corresponding to the adsorbent/adsorbate system drops linearly rather than logarithmically^41^.

### Adsorption thermodynamics

Adsorption thermodynamics is a term used to refer to various temperature-dependent parameters such as Gibbs free energy, entropy, enthalpy, etc. The adsorption thermodynamics can provide an insight about the spontaneity and feasibility of adsorption process^46^. Three thermodynamic parameters, which were calculated across a temperature from 25 to 45 °C, are: Gibbs free energy, enthalpy and entropy, were investigated in this section. The investigated temperature range in the present study is environmentally relevant for the Middle East and North Africa (MENA) area.

The Gibbs free energy $\Delta G^{0}$ and the adsorption equilibrium constant (K_eq_) were calculated using Eqs. (6, 7):

6 $$\Delta G^{0} = - RT\;\ln \;K_{eq}$$

7 $$\Delta G^{0} = \Delta H^{o} - T\Delta S^{o}$$

8 $$K_{eq} = \frac{{q_{e} }}{{c_{e} }}$$

where universal gas constant is denoted by *R* (8.314 J/K·mol), temperature is denoted by *T* (Kelvin), $C_{e}$ and *q*_*e*_ are the final concentration of phosphate in water (mg/L) in addition to equilibrium adsorption capacity of adsorbent (in mg/g), respectively.

*K*_*eq*_ and the enthalpy as well as entropy can be correlated using the linearized form of the Gibbs Helmholtz equation:

9 $$ln\;K_{eq} = \frac{{\Delta S^{0} }}{R} - \frac{{\Delta H^{0} }}{RT}$$

Plotting ln *K*_*eq*_ vs $\frac{1}{T}$ gives a straight-line relationship. The y-intercept as well as slope of the correlation are: $\frac{{\Delta S^{0} }}{R}$ and $\frac{{\Delta H^{0} }}{R}$, respectively.

## Results and discussion

### BC characterization

#### XRD, SEM and XPS characterization

**Figure 1:**
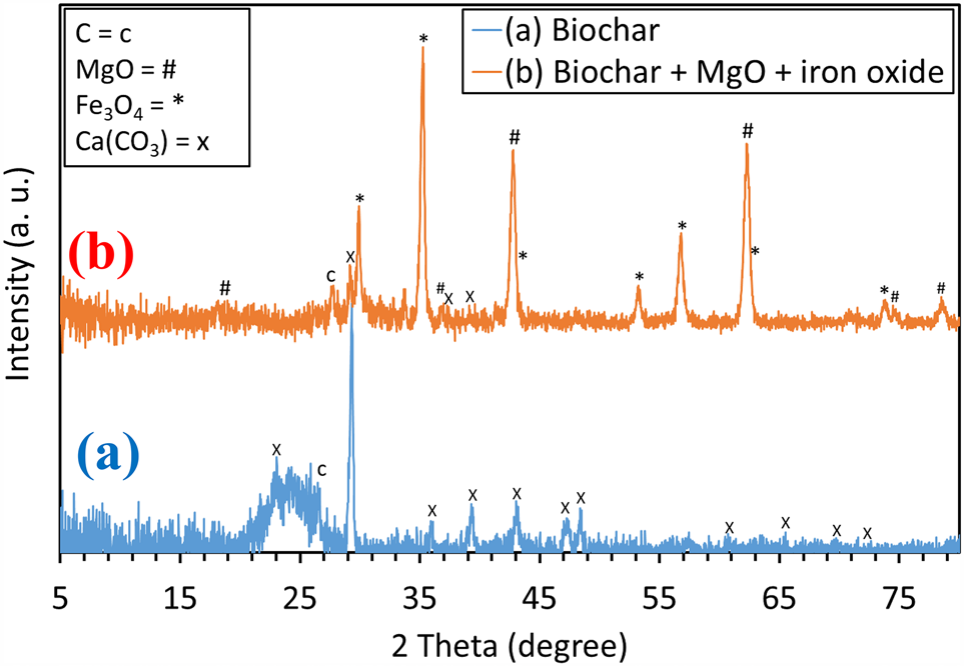
XRD patterns of BC (**a**); (**b**) BC doped MgO and iron oxide synthesized via hydrothermal process.

Figure 1 depicts XRD patterns of the as-prepared BC at 600 °C (a) and BC doped with MgO and Fe_3_O_4_ (b). In Fig. 1a, there are two phases of amorphous carbon (JCPDS No. 01-082-9929) and calcite (CaCO_3_) (JCPDS No. 01-078-3262). However, in Fig. 1b, there are four phases of carbon (JCPDS No. 01-082-9929) and calcite/calcium carbonate (CaCO_3_) (JCPDS No. 01-078-3262), Fe_3_O_4_ magnetite with high crystallinity phase (JCPDS No. 01-082-9929), and MgO with high crystallinity phase (JCPDS No. 04-016-6860).

**Figure 2:**
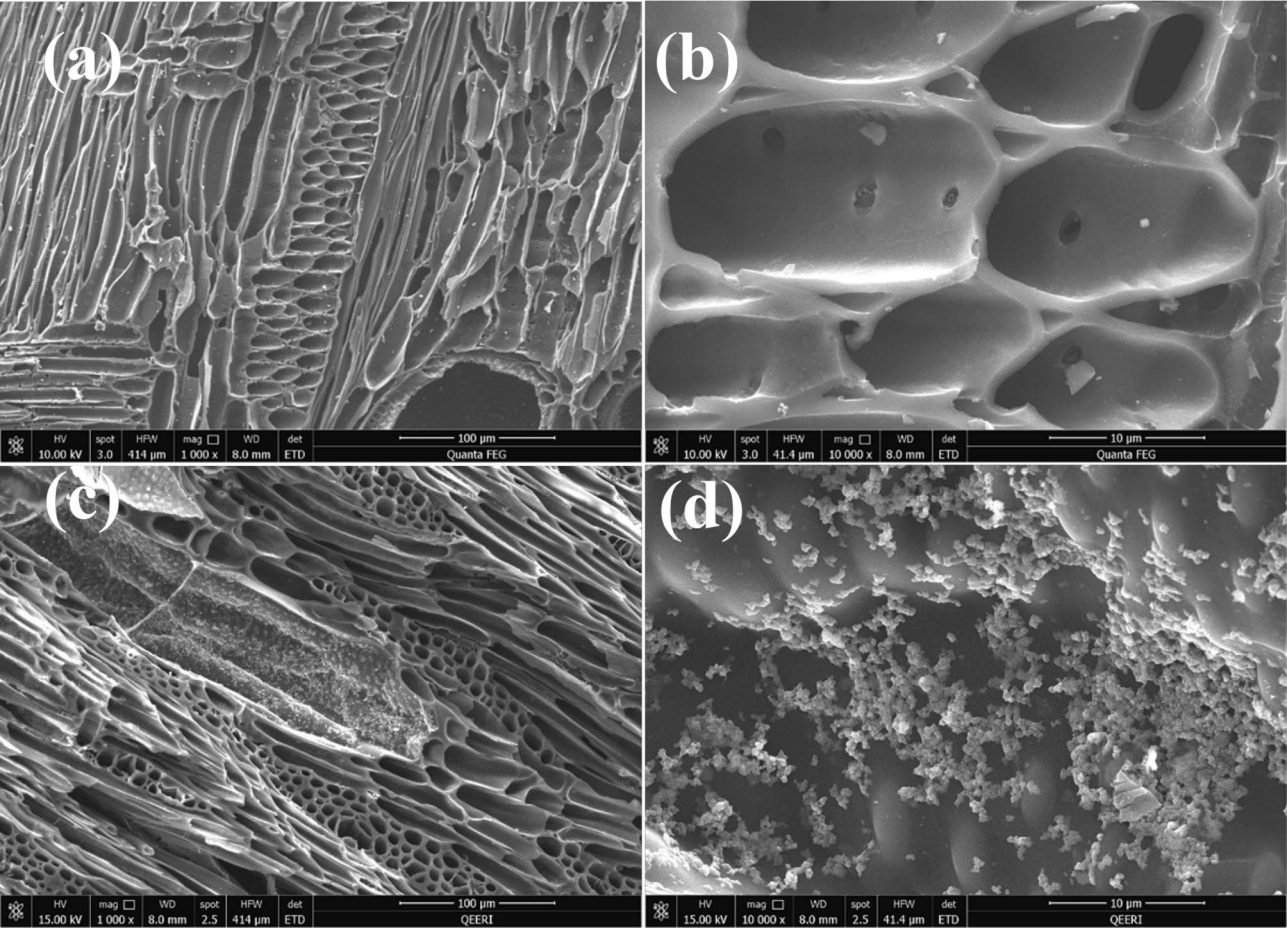
SEM images of biochar (**a**) and (**b**); biochar doped with Fe_3_O_4_ and MgO synthesized via hydrothermal process (**c**) and (**d**).

**Figure 3:**
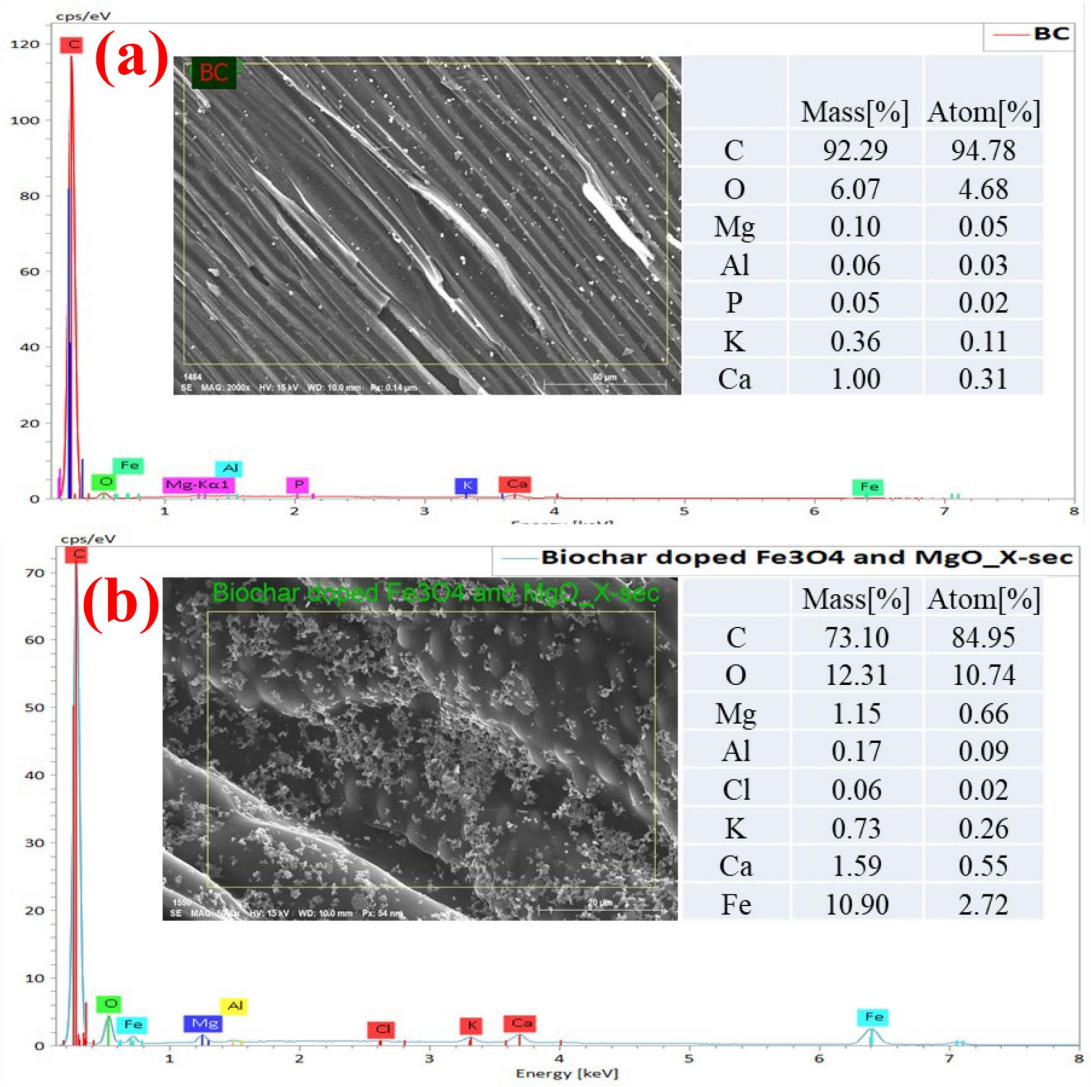
EDS analysis of BC (**a**); BC doped Fe_3_O_4_ and MgO synthesized via hydrothermal process (**b**).

SEM images of BC prepared at 600 °C show widely developed micropores structure, as shown in Fig. 2a,b, and after doping with MgO and Fe_3_O_4_, there is a regular distribution of MgO and Fe_3_O_4_ particles over the BC surface, as shown in Fig. 2c,d. EDS elemental analysis of raw BC and BC doped with Fe_3_O_4_ and MgO was shown in Fig. 3. As seen, mass percentages of the iron, magnesium and oxygen in the modified BC were higher than that of the unmodified BC due to the incorporation of Fe_3_O_4_ and MgO in the modification process.

XPS was employed to investigate surface elemental in addition to chemical states of BC prior to and post PO_4_^3−^ adsorption. To reveal the chemical information, the XPS measurements are performed, and the comparison of the main elements among the 3 samples (S1 is BC, S2 is BC doped with MgO and Fe_3_O_4_, and S3 is S2 after adsorption of phosphate is listed in Fig. 1), with the corresponding deconvolution results given in the supporting information (Figs. S1–S3).

**Figure 4:**
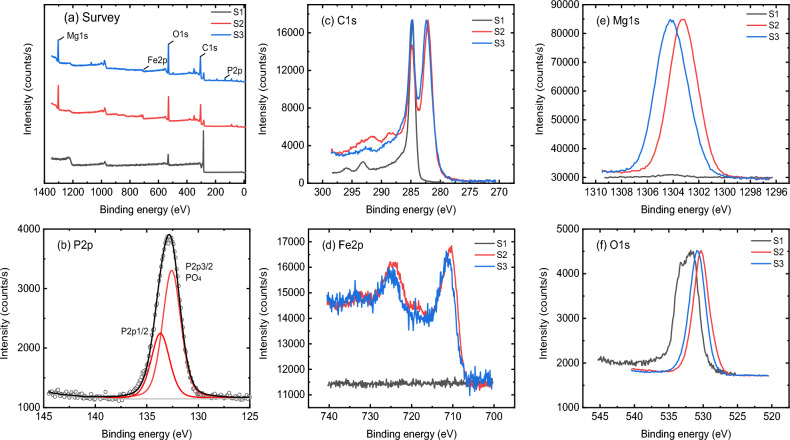
Comparison of the relevant elements among the BC samples: (**a**) the survey spectra; (**b**–**f**) P2p, C1s, Fe2p, Mg1s, and O1s, respectively. The intensities are normalized to the maximum for a better comparison.

Figure 4a presents the survey spectra where only C1s and O1s are observed in Sample 1, extra Fe2p and Mg1s are given for Sample 2 and 3, while Sample 3 exhibits an additional signal of P2p (see the corresponding atomic ratio of all elements in Table 3). The fitting of P2p in Fig. 4b presents the phosphate (PO_4_^3−^) signal (~ 6% in atomic ratio) with the P2p3/2 at 132.6 eV, indicating a successful adsorption of the PO_4_^3−^ ions from the target solution. As shown in Fig. 4c, the C1s of Sample1 has the typical Biomass structure with a major C–C bond and weak oxidation-related signals (also see fitting in Fig. S1 in the SI section). Some K2p signal was observed, relating to residuals during the preparation process. After doping with Fe_3_O_4_ and MgO, an equally intense signal appears at 282.1 eV, relating to the metal carbide bond^47–49^, probably formed during the annealing process (see the sample preparation section). Figure 4d,e presents the metal signals Fe2p and Mg1s for Sample 2 and Sample 3, where a slight shift of Mg1s is observed, while no clear variation in profile is given in the case of Fe2p. The fitting of Mg1s in Figs. S2 and S3 shows 3 components locating at around 1302.6 eV and 1303.9 eV, assigned to Mg–C, Mg–O, and another signal of Mg–OH at 1305.0 eV specifically observed in Sample 3, contributing to the binding energy shift observed in Fig. 1e^50^. Meanwhile, the fitting of Fe2p reveals the Fe–O-related FeII and FeIII states, with Fe2p3/2 locating at around 711.7 eV and 709.8 eV, respectively^51^. The evolution of O1s is given in Fig. 4f, where an organic C–O and C=O bond are initially observed for the raw biomass sample 1, both of which were strongly buried by the metal-oxidation signal as well as the metal-OH bond, locating at around 528–530 eV and 531 eV, respectively. (as shown by the deconvolution of O1s in Figs. S1, S2, and S3).

**Table 3:** Atomic ratio of all elements calculated with the intensity of the high-resolution spectra.

Atomic ratio (%)	C	O	Fe	Mg	P
S1	87.8	12.2	–	–	–
S2	28.7	46.4	3.1	21.8	–
S3	34.1	43.3	1.1	15.6	5.9

#### Zeta potential

**Figure 5:**
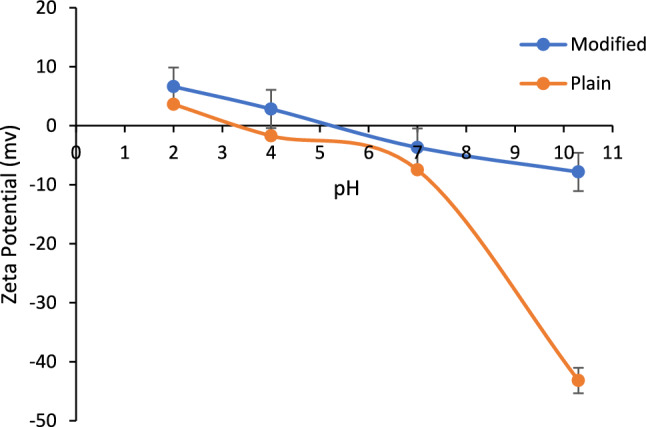
Zeta potential of plain and modified BC versus pH of the aqueous solution.

The surface charge of the plain and modified BC was plotted and depicted in Fig. 5. Both plain and modified BC showed positive zeta potential values at low pH obviously due to the protonation of BC with H^+^ ions added during pH adjustment by HCl. The point of zero electric charge (PZC) of modified BC was found to correspond to about pH of 5.3 whereas the PZC of plain BC was at about 3.4. This was observed to be in good agreement with the PZC of BC modified with iron for the removal of PO_4_^3−^ from water which was reported by Yang to be at pH 6^52^. The decrease in solution pH showed a decrease in zeta potential due to the adsorption of OH^−^ by BCs during pH adjustment with NaOH. Moreover, as seen in Fig. 5, the modification of the BC by the addition of Fe_3_O_4_ and MgO has the effect of shifting PZC as well as reducing the negative surface charge of the modified BC which will eventually increase the attraction forces between the adsorbent and PO_4_^3−^ ions in water. This was observed to be in good agreement with results reported in literature^24,53,54^.

#### BET surface area

**Figure 6:**
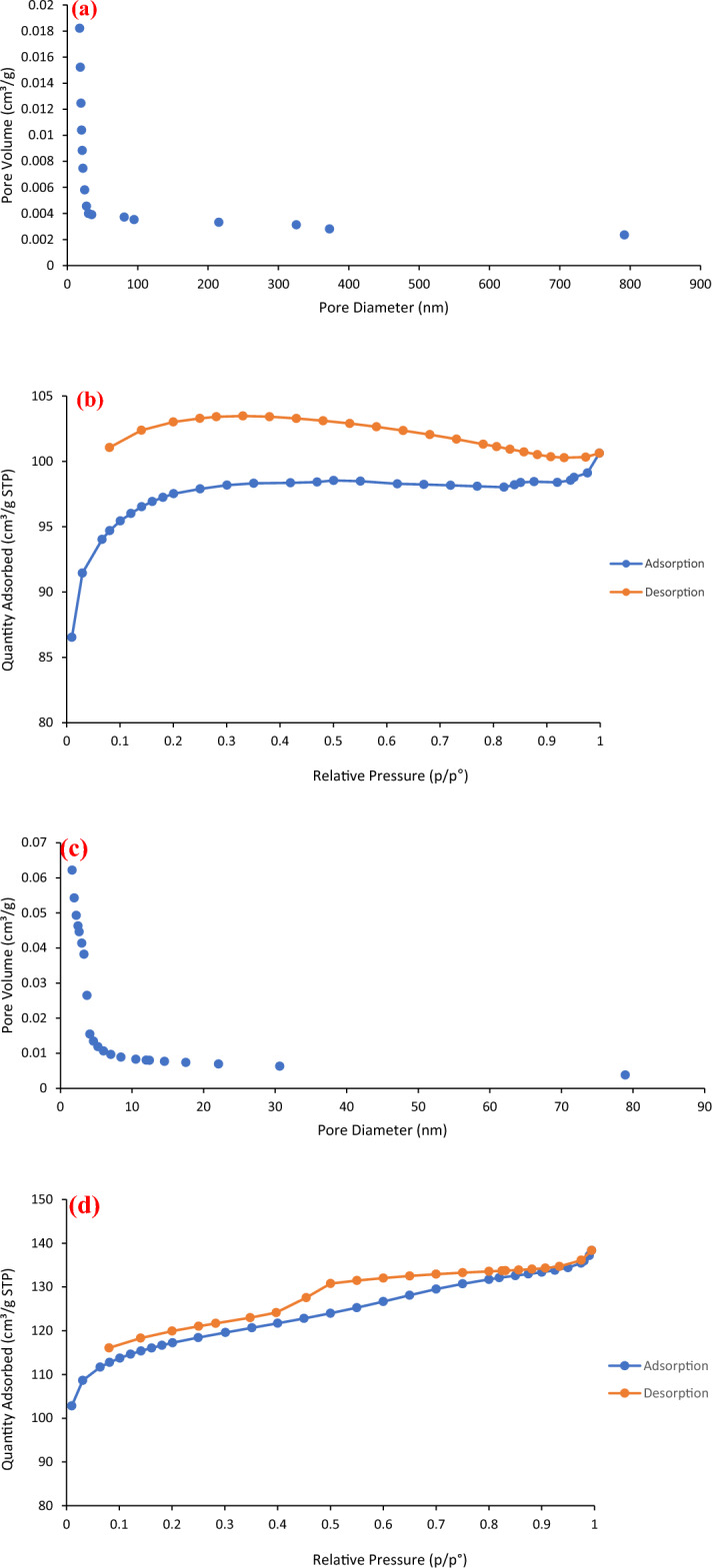
(**a**) Pore size distribution curve of BC, (**b**) N_2_ adsorption/desorption isotherm of pure BC, (**c**) Pore size distribution curve of modified BC and (**d**) N_2_ adsorption/desorption isotherm of modified BC.

The pore distribution and the BET surface area corresponding to the plain and modified BC was evaluated using BJH method. Figure 6a–d show the pore volume at different pore diameters as well as the N_2_ adsorption/desorption isotherms of pure and modified BC. The figure showed that the modified BC had micropores and the majority of the pores ranging between 1.6 and 80 nm. Moreover, the average surface area corresponding to the plain and modified BC was found to be 322 and 394 m^2^/g. The modification of the BC was found to increase the porosity and BET surface area by more than 20% after the modification by impregnation with iron oxide and magnesium oxide.

The enhancement in the surface area of BC after doping with Fe_2_O_3_ and MgO was found to be in good agreement with the studies cited in literature for the BC modification with various positively charged metals such as: magnesium^15^, calcium^17^, lanthanum^13,16^ and aluminum^14^ which were reported to show higher surface area and enhanced removal of phosphate from water compared with plain BC^53^. For instance, the introduction of magnesium into BC was reported to increase the surface area from 26.3 to 170.2 m^2^/g due to the inhibition of the formation of volatile compounds and tar during the pyrolysis^15,55^. Moreover, the increase in the BET surface area of BC modified with Mg was reported to take place due to the characteristic crystal forming nature in Mg compounds which produced rougher surfaces^14,56^. Likewise, the preparation of BC modified with iron oxide was reported to increase the surface area by 340%^49,50^ due to the release of volatiles from BC matrix during the synthesis process. Furthermore, the enhancement in the surface area of BC after modification with metals can be attributed to the occurrence of some changes taking place during pyrolysis process which are initiated by the incorporation of metal salts into biomass^20^. This results in the formation of porous structures within the BC due to the presence of metal precipitate template which inhibits the aggregation of nearby carbon atoms^57–59^.

### Adsorption experiments

#### Effect of doping composition, adsorption time, BC dose and shaking speed

**Figure 7:**
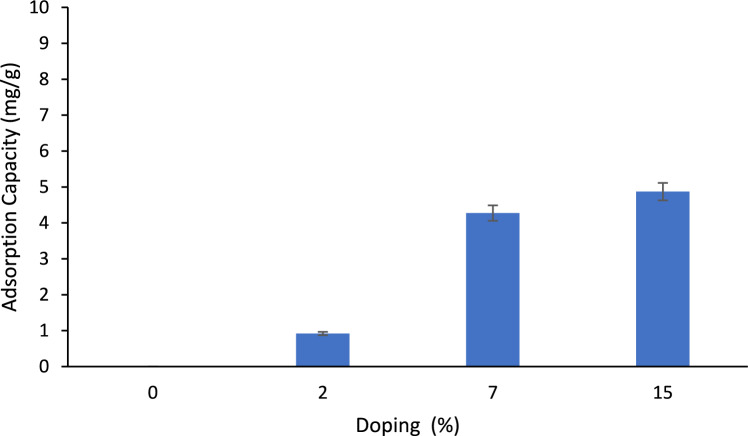
Removal efficiency of PO_4_^3−^ on BC at various Fe_3_O_4_ and MgO loadings; PO_4_^3−^ concentration: 10 ppm, BC loading: 2 g/L, pH: 8.5, agitation speed: 150 RPM, adsorption time: 4 h.

Figure 7 shows the adsorption capacity of unmodified BC as well as modified BC at 2, 7 and 15 wt.% loading of iron and magnesium oxides. It was found that the adsorption of unmodified BC was negligible whereas the modification of BC with Fe_3_O_4_ and MgO increased the adsorption capacity significantly. As seen, the adsorption capacity of modified BC at 7% and 15% metal oxides loading were 4.27 ± 0.14 and 4.87 ± 0.21 mg/g, respectively. As observed, the adsorption capacities did not increase significantly; hence, the optimum Fe_3_O_4_ and MgO loading which will be carried out in the present work is 7%. Nazal et al.^60^ prepared 4 activated carbon (AC) and 4 carbon nanotubes (CNTs) adsorbents at various nickel oxide doping compositions (1, 5 and 10%). It was reported that the highest doping composition of nickel oxide in both AC and CNTs was 5%. The reduction or stabilization in the adsorption capacity upon the increase in the doping composition beyond 5% was attributed to the agglomeration of nickel oxide nanoparticles which eventually reduced the available active adsorption sites on the AC and CNTs^60^.

**Figure 8:**
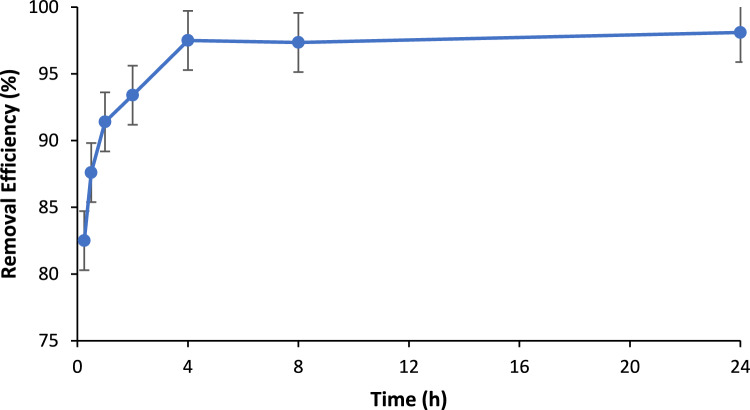
Removal efficiency of BC vs contact time: BC loading: 2 g/L, PO_4_^3−^ concentration: 10 ppm, pH: 8.5, agitation speed: 150 rpm.

The impact of adsorption time on PO_4_^3−^ removal efficiency using BC was carried out by changing the contact time between 1 min and 24 h while fixing all the other parameters (initial PO_4_^3−^ level of 10 ppm and BC loading of 2 g/L). Figure 8 depicts the impact of contact time on removal efficiency. As shown, it was observed that the adsorption process was very fast and the phosphate removal value of 82.5% was reached only after 30 min of adsorption, while the removal efficiency after 4 h of adsorption was 97.5%. The rapid removal efficiency in short contact time is credited to high BC’s surface area. As seen in Fig. 8, a removal efficiency of 97.5% was achieved after 4 h adsorption time; hence, the optimized adsorption time of 4 h will be carried out throughout the rest of the present work. Keeping BC for longer adsorption time was not going to increase the removal efficiency significantly obviously due to satiation of adsorption sites at BC surface.

**Figure 9:**
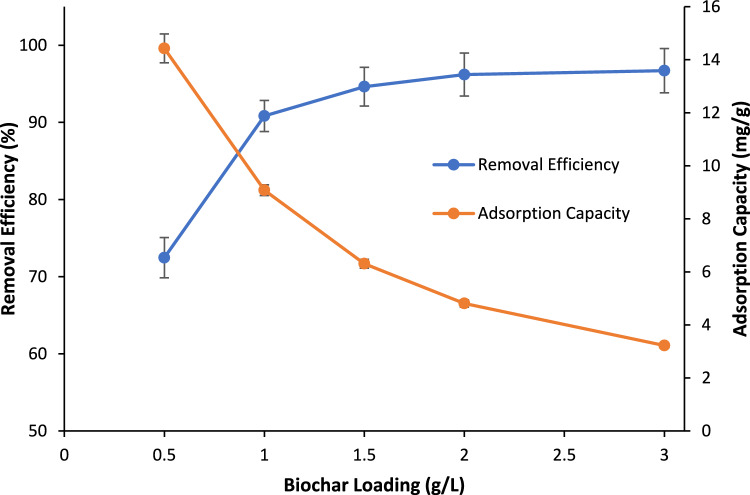
Removal efficiency as well as adsorption capacity of PO_4_^3−^ on BC vs BC loading (g/L); PO_4_^3−^ concentration: 10 ppm, pH: 8.5, adsorption time: 4 h, agitation speed: 150 RPM.

In the present work, the BC loading was varied between 0.5 and 3 g/L. Figure 9 depicts the effect of BC loading on removal efficiency as well as adsorption capacity. As observed, the removal efficiency was found to increase along with the increase in the BC loading until it reaches 96.2% at a BC loading of 2 g/L. Moreover, the raise in adsorbent loading was found to lower adsorption capacity from 14.4 mg/g at BC loading of 0.5 mg/L to 3.2 mg/g at BC loading of 3 mg/L. This can be correlated to take place due to availability of more active adsorption sites upon the increase in the adsorbent loading in solution. These active adsorption sites were reported in literature to remain unsaturated and reduce the adsorption capacity at constant adsorbate loadings^61,62^.

**Figure 10:**
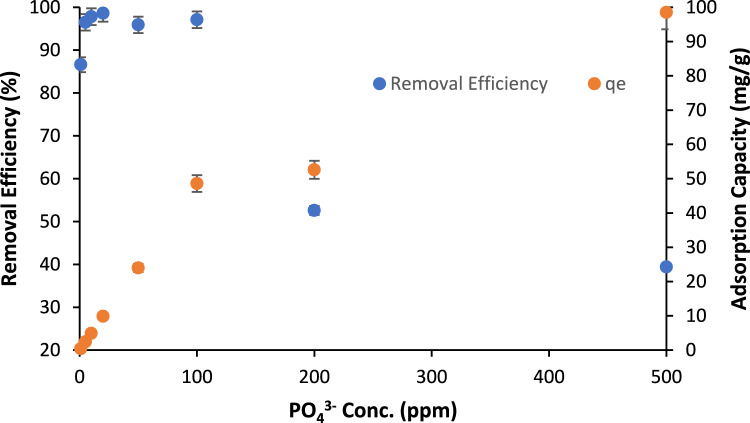
Removal efficiency of PO_4_^3−^ on BC vs PO_4_^3−^ concentration (ppm): BC loading: 2 g/L, pH: 8.5, shaking speed: 150 RPM, sorption time: 4 h;

Figure 10 shows the removal efficiency of PO_4_^3−^ and the adsorption capacity (q_e_) at various PO_4_^3−^ concentrations while maintaining the BC loading at 2 g/L and adsorption time at 4 h. It was observed that increasing the PO_4_^3−^ concentration results in reducing the removal efficiency which was ascribed to the occupation of available adsorption sites on BC with adsorbed phosphate. Conversely, the increase of the PO_4_^3−^ concentration was found to elevate adsorption capacity from 0.4 mg/g at PO_4_^3−^ concentration of 1 ppm up to 98.5 mg/g at PO_4_^3−^ concentration 500 ppm. As seen in Table 4, the performance of the modified BC was found to outperform some of the sorbents employed for removal of PO_4_^3−^ from water and reported in literature^16,24,63–69^. For instance, Jing et al.^63^ developed a BC modified with Fe^3+^ from the cotton stalk to remove PO_4_^3−^ from water. Despite testing the BC in low PO_4_^3−^ concentration (20 mg/L), the modified adsorbent exhibited a low adsorption capacity of 0.963 mg/g and long adsorption time of 24 h. likewise, Chen et al.^64^ synthesized a magnetic BC modified with Fe^2+^ and Fe^3+^ from agricultural biomass waste for the sake of removing PO_4_^3−^ from water. The magnetic biochar was tested with low PO_4_^3−^ concentration of 2.4 mg/L and high BC loading of 6.25 g/L. Nevertheless, the adsorption capacity was very low (1.24 mg/g).

**Table 4:** Comparison between the performance of the modified BC adsorbent in the present work and some of the BC modified with metals used for PO_4_^3−^ removal from water from literature.

Metal	BC loading (g/L)	PO_4_^3−^ conc.	Main findings	References
Fe^3+^	2	20 mg/L	Max q_*e*_: 0.963 mg/g Equilibrium time: 24 h	^63^
Fe^2+^ and Fe^3+^	6.25	2.4 mg/L	Max *q*_*e*_: 1.24 mg/g	^64^
Fe^2+^ and Fe^3+^	10	150 mg/L	Max *q*_*e*_: 28 mg/g Equilibrium time: 5 h	^65^
Mg and Al	2	50 mg/L	Max *q*_*e*_: 74.47 mg/g Equilibrium time: 5 h	^24^
Fe/Zn/Cu	2	50	Max* q*_*e*_: 45 mg/g Equilibrium time: 4 h	^67^
Mg/Al	2.5	50	Max* q*_*e*_: 81.8 mg/g Equilibrium time: 40 min	^69^
La	2	400	Max *q*_*e*_: 46.4 mg/g Equilibrium time: 10 h	^68^
Ce/Fe_3_O_4_	1	75 mg/L	Max *q*_*e*_: 18.7 mg/g Equilibrium time: 3 h	^16^
Fe_3_O_4_/MgO	2	500	Max *q*_*e*_: 98.5 mg/g Equilibrium time: 4 h	Present work

#### Adsorption isotherms

**Figure 11:**
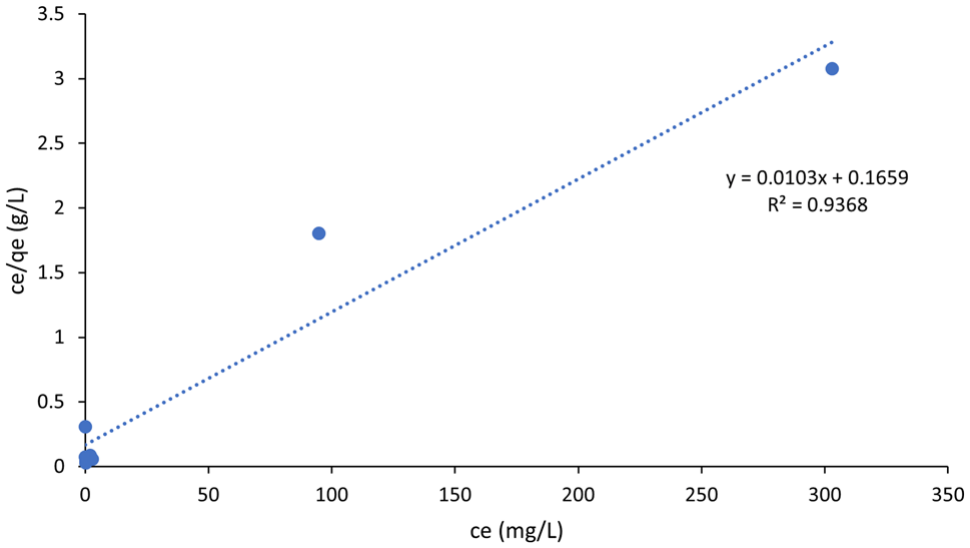
Linear fitting of experimental adsorption data to Langmuir adsorption isotherm for the removal of PO_4_^3−^ by BC. pH: 8.5, BC dosage: 2 g/L and adsorption time: 4 h.

The adsorption isotherms were also discussed in the present work by linear fitting the experimental data to four models which are: Langmuir, Freundlich, Temkin as well as Sips isotherms. Figure 11 shows the linear curve fitting of experimental adsorption data using Langmuir isotherm model. Moreover, parameters related to each of the investigated models were listed in Table 5. The best fit of the four isotherm models was decided by comparison between correlation coefficient (*R*^2^). As depicted in Fig. 11 and Table 5, adsorption of PO_4_^3−^ onto BC can be described by Langmuir isotherm model. Owing to the highest *R*^2^ (0.937), the best fitting of the Langmuir model amongst the four isotherm models confirms the monolayer adsorption of PO_4_^3−^ onto the BC surface. This means that the adsorption of PO_4_^3−^ onto the surface of the BC assumes a uniform distribution of binding energies^70^. These findings were observed to correlate well with results cited in literature which described the adsorption of PO_4_^3−^ onto the modified BC to be best fitted by Langmuir isotherm model^65–68^. Moreover, the favorability of the adsorption of PO_4_^3−^ onto BC was determined by estimating the separation factor (*R*_*L*_) as shown in Eq. (5). The value of the separation factor describing the adsorption of PO_4_^3−^ onto BC was found to fall between 0 and 1 (0.00945–0.956) which indicates the favorability of the adsorption process^43^.

**Table 5:** Isotherm adsorption parameters corresponding to Langmuir, Freundlich, Temkin as well as Sips models.

Model	Parameters		R^2^	SSE
Langmuir	*q*_*m*_	78.1 mg/g	0.937	1170
	*K*_*L*_	0.346 L/mg		
	$R_{L}$	0.00945–0.956		
Freundlich	*n*	1.94	0.702	28,600
	*K*_*f*_	8.10		
Temkin	*b*	227 J/mol	0.900	826
	*K*_*t*_	8.02 L/g		
Sips	*n*	1.79	0.681	30,600
	*Ks*	9.88 L/g		

Furthermore, based on an *R*^2^ value of 0.900, Temkin isotherm model is the second-best fit model for experimental adsorption data corresponding to the adsorption of phosphate with biochar system. The Temkin model takes into assumption that adsorption heat corresponding to molecules participating in the adsorption process declines linearly in consequence of the interaction between adsorbate as well as adsorbent. Moreover, the Temkin isotherm model describes an adsorption system with consistent distribution of bonding energies which is capped by a maximum value^71,72^. The value of Temkin constant can be used to give an indication about the kind of sorption mechanism occurring (physical, chemical, or a combination). In the present work, the value of Temkin constant corresponded to 227 J/mol or 0.0543 kcal/mol which is below 1 kcal/mol. An adsorptive system with a Temkin constant less than 1 kcal/mol refers to physical sorption^73^. Hence, the adsorption of PO_4_^3−^ onto BC can be described as physical sorption.

#### Adsorption kinetics

**Figure 12:**
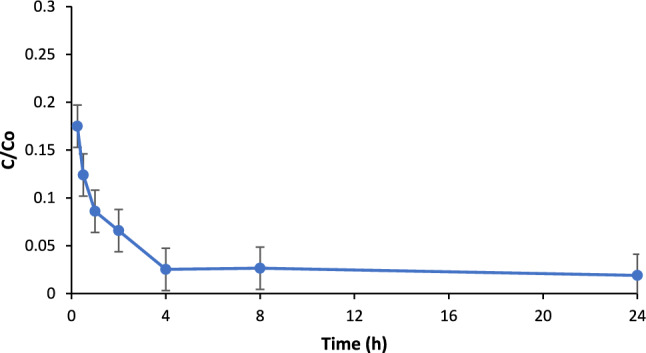
Nominal equilibrium concentration of PO_4_^3−^ vs adsorption time with modified BC; BC loading: 2 g/L, pH: 8.5, shaking speed: 150 rpm.

The adsorption kinetics of the PO_4_^3−^ on BC was found to be quite fast. Figure 12 shows the nominal equilibrium concentration of phosphate in the feed solution over the adsorption time. As seen, the modified BC removed more than 80% of the PO_4_^3−^ in less than 30 min and almost complete removal was achieved in 4 h. In the present work, the experimental data were fitted into four kinetic models which are: pseudo 1st order, pseudo 2nd order, Elovich as well as intra particle diffusion models. As seen in Table 6 and based on the value of coefficient of determination (*R*^2^ = 0.952), pseudo 2nd order model better describes the adsorption of PO_4_^3−^ onto BC. This can be also confirmed by comparing the qe value corresponding to pseudo 2nd order model which was closer to qe (experimental) value of 4.91 mg/g than that of pseudo 1st order model. On the other hand, pseudo 1st order and intra particle diffusion models showed low *R*^2^ values corresponding to 0.675 and 0.605, respectively. This can be used to denote that the absorption of PO_4_^3−^ onto BC cannot be accurately represented by the above-mentioned models. When comparing the Elovich model to pseudo 1st order as well as intra particle diffusion models, it seems that Elovich model can better represent the adsorption of PO_4_^3−^ onto BC system based on the* R*^2^ value of 0.874. The Elovich kinetic model can be used to estimate number of adsorption sites in BC by calculating the reciprocal of $\beta$. The required time to adsorb one-half of PO_4_^3−^ from solution onto BC is also known as half-life. Equations (3 and 4) were used to determine half-life time using pseudo 1st as well as 2nd order parameters. In the present work, the calculated half-life time using pseudo first and second order parameters were 5.41 min and 0.0684 min (or 4.11 s), respectively. Since the adsorption of PO_4_^3−^ onto BC was found to follow pseudo second order model, the half-time which will be considered in the present work will be based on the pseudo second order parameters. The short half-life time calculated in the present work can be used to indicate high rate of adsorption process. This can be manifested by reducing the needed adsorption time in water treatment facilities, which will lower the operational costs.

**Table 6:** Kinetic models parameters corresponding to the investigated adsorption of PO_4_^3−^ by BC.

*q*_*e*_ (experimental)		4.91 mg/g
Pseudo-first order	*q*_*e*_ (model)	4.74 mg/g
	*k*_*1*_	0.128 $\frac{1}{min}$
	*R*^2^	0.675
Pseudo-second order	*q*_*e*_ (model)	4.92 mg/g
	*k*_*2*_	2.97 $\frac{g}{{\frac{mg}{{min}}}}$
	*R*^2^	0.952
Elovich	$\alpha$	5.83 $\frac{mg}{\frac{g}{min}}$
	$\beta$	7.03 × 10^8^ g/mg
	*R*^2^	0.874
Intra-particle diffusion	*k*_*IP*_	0.0191 $\frac{mg}{{\frac{g}{{min^{0.5} }}}}$
	*c*	4.35 mg/g
	*R*^2^	0.605

#### Effect of feed temperature and adsorption thermodynamics

**Figure 13:**
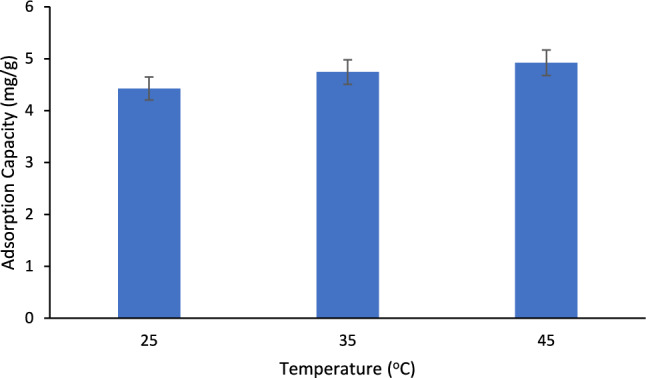
Adsorption capacity vs the solution temperature for the adsorption of PO_4_^3−^ with BC; BC loading: 2 g/L, pH: 8.5, agitation speed: 150 rpm.

In the present work, the influence of aqueous solution’s temperature on adsorption performance was investigated by determining adsorption capacity at three solution temperatures which are: 25, 35 and 45 °C. The influence of temperature on adsorption is depicted in Fig. 13 which shows a slight enhancement in adsorption capacity along with the solution temperature. Owing to increased mass transfer rates between PO_4_^3−^ and BC at higher temperatures, the enhancement in the adsorption performance was observed to correlate well with work cited in literature^65,66,68^. For instance, the influence of temperature on performance of lanthanum-loaded BC, which were developed from oak chips, during the removal of PO_4_^3−^ from water was studied at four temperatures (15, 25, 35 and 45 °C). It was observed that adsorption capacity of modified BC rose from 4 to 60 mg/g along with the increase in the temperature due to the energetic thermal motion which enhanced the likelihood of collisions between PO_4_^3−^ and adsorption sites^68,74^. Likewise, the BC prepared from wood and rice husk and doped with Fe(II) and Fe(III) ions was reported to show enhanced adsorption performance (from 6 to 12 mg/g) towards PO_4_^3−^ when the solution temperature was increased from 15 to 55 °C^65^.

The surge in adsorption along with the temperature can be ascribed to the enhanced dispersibility of BC along with higher diffusion of PO_4_^3−^ ions^82^. For instance, the BC prepared from banana and modified with iron oxide and cobalt^75^ for removal of amoxicillin from water showed an increase in adsorption capacity along with temperature which designates that adsorption was endothermic and that the elevated temperatures provided higher energy to amoxicillin molecules to reach the BC pores and adsorb more efficiently^76,79^. The same behavior was also observed with Hanane et al.^77^ who prepared BC from date palm modified with iron for the removal of ofloxacin from water. The adsorption was cited to rise along with temperature, which supports the endothermic characteristic of the adsorption process, due to the increase in the diffusion rate of ofloxacin to reach the pores of modified BC.

The adsorption thermodynamic parameters related the sorption of PO_4_^3−^ onto BC such as enthalpy (∆H^0^), entropy (∆S^0^) and Gibbs free energy (∆G^0^) can be determined using Eqs. (6–8). The calculated thermodynamic parameters within a temperature range that starts from 25 °C until 45 °C (298.15–318.25 K) are shown in Table 7. As seen, Gibbs free energy values corresponding to adsorption of PO_4_^3−^ ions onto BC ranged between − 3.17 and − 8.92 kJ/mol following the rise in the elevation of solution temperature from 25 to 45 °C. The negative sign of the calculated Gibbs free energy throughout investigated temperatures indicates spontaneity of adsorption process. Furthermore, the state of disorder or entropy corresponding to the adsorption of PO_4_^3−^ ions onto BC was calculated using Eq. (8) and reported to be 0.287 kJ/mol. The positive sign of the entropy indicates the rise in the system’s degree of disorder. The calculated enthalpy change for the adsorption of PO_4_^3−^ ions onto BC was found to be 82.5 kJ/mol. The positive sign of the enthalpy change was reported in literature to denote endothermic reactions. This was observed to correlate well with work cited in literature^78–80^. For instance, the adsorption of PO_4_^3−^ ions with corn BC, which was modified with Mg, was reported to be endothermic^79^. Likewise, the adsorption of PO_4_^3−^ onto BC prepared from *Undaria* *pinnatifida* roots and modified with MgFe_2_O_4_ ions was reported to increase along with temperature and the adsorption process was endothermic too^80^.

**Table 7:** Thermodynamic parameters (entropy, enthalpy as well as Gibbs free energy) corresponding to the adsorption process of PO_4_^3−^ onto BC at different temperatures; BC loading: 2 g/L, shaking speed: 150 rpm; and pH: 8.5.

Temperature (°C)	$\Delta S^{^\circ }$(kJ/mol)	$\Delta H^{^\circ }$(kJ/mol)	$\Delta G^{^\circ }$(kJ/mol)
25	0.287	82.5	− 3.17
35			− 6.05
45			− 8.92

#### Effect of pH

**Figure 14:**
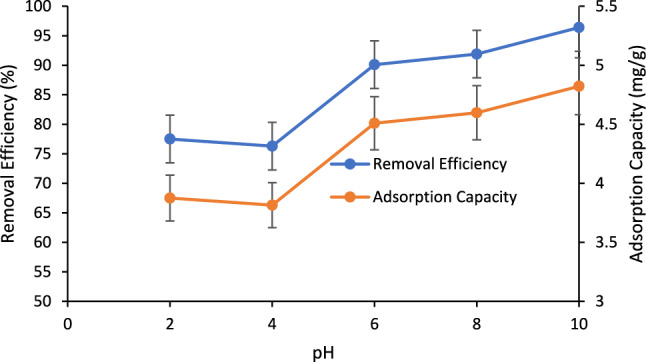
Removal efficiency of PO_4_^3−^ ions on BC vs pH; phosphate concentration: 10 ppm; BC loading: 2 g/L; shaking speed: 150 RPM, residence time: 4 h.

The influence of solution pH (2, 4, 6, 8 and 10.3) on phosphate adsorption was investigated and depicted in Fig. 14. The change in the removal efficiency as well as adsorption capacity of the system indicates its pH-dependency. The increase in the solution pH from 2 till 10.3 increased the removal efficiency from 77.5 to 96.4% and adsorption capacity from 3.8 to 4.8 mg/g.

**Figure 15:**
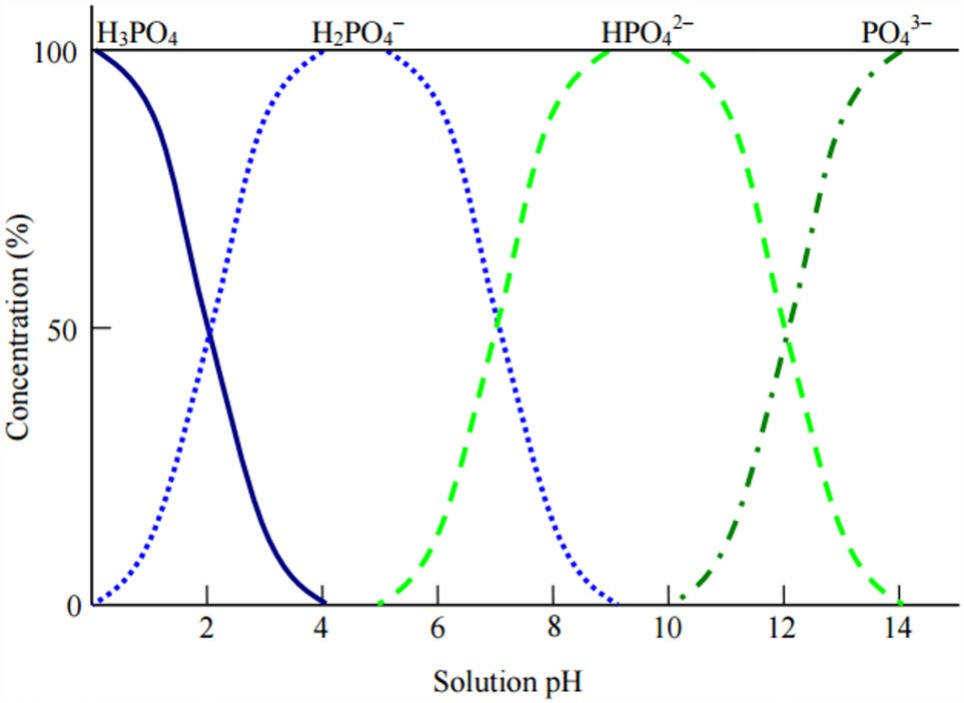
Phosphate species in water at different solution pH^81^.

The enhancement in the adsorption of PO_4_^3−^ can be attributed to an increase of degree of dissociation of PO_4_^3−^ ions in water with increasing pH (Fig. 15) which tends to increase electrostatic interaction forces between negatively charged phosphate ions and positively charged BC surface. This can be confirmed by referring to Fig. 5 which shows the modified BC’s pH_PZC_ of about 5.3 and the reduced overall negative surface charge in the modified BC at pH > 5.3. The rise in removal efficiency along with the surge in solution pH was also investigated in literature when the BC modified with Mg was tested at a pH range of 6–10 and was reported to increase along with pH and outperform the plain BC^79^. This was attributed due to the presence of Mg which increased the chelation between PO_4_^3−^ ions and BC. The authors attributed the enhancement in the adsorption performance of modified BC due to the physical and chemical adsorption as opposed to the physical adsorption only corresponding to plain BC.

#### Effect of co-existing ions on the adsorption of by BC

**Figure 16:**
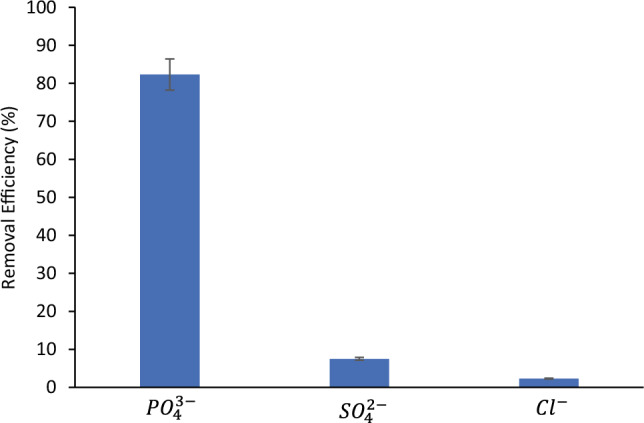
Removal efficiency of PO_4_^3−^, SO_4_^2−^ and Cl^—^ions with BC. The concentration of each ion is 10 ppm; BC loading: 2 g/L; agitation speed: 150 RPM, adsorption time: 4 h.

The influence of co-existing ions on the adsorption of PO_4_^3−^ ions by BC was studied in the present work with adsorption experiments with 10 ppm multicomponent solution of phosphate, chloride and sulphate ions. Figure 16 shows the phosphate removal with BC from multicomponent solution was as high as 82%. This finding confirms that modified BC can be used for efficient phosphate removal even from multicomponent solutions containing different anions. Conversely, the adsorption capacity of modified BC towards phosphate ions was found to slightly reduce from 4.27 to 4.12 mg/g (3.5% reduction) obviously due to the competition between PO_4_^3−^ ions and the other co-existing ions in solution to occupy vacant adsorption positions in BC.

The reason behind the maintenance of significant adsorption capacity corresponding to PO_4_^3−^ compared with other competing ions in water can be attributed to the electronegativity of the competing ions. For instance, Kang^25^ tested the effect of competing anions on the removal efficiency of BC modified with iron towards PO_4_^3−^ ions in water and observed that the modified BC maintained their performance while other anions such as HCO_3_^−^ and SO_4_^2−^ showed a reduction in adsorption capacities exceeding 42% and 35%, respectively. The authors attributed the maintenance of adsorption capacity towards PO_4_^3−^ and reduction with other anions to take place due to the lower electronegativity corresponding to PO_4_^3−^ compared with other anions. The electronegativity of the investigated anions was found to follow the order of: PO_4_^3−^ (χ = 2.19) < HCO_3_^−^ (χ = 2.55) < SO_4_^2−^ (χ = 2.58)^82^. The authors also reported that ligand bonding between BC and anions in water is better for low electronegativity anions. On the other hand, non-specific anion exchange is more pronounced for anions with high electronegativity^82^. Hence, the higher the electronegativity is, the higher the inhibition order is. This indicates that the main interaction between the biochar and PO_4_^3−^ ions in the present work can be attributed to the ligand exchange^25^.

The performance of the modified BC in the present work was observed to outperform the work reported by Almanassra et al.^22^ who tested the removal of PO_4_^3−^ ions from water in the presence of other competing ions (PO_4_^3−^, SO_4_^2−^, NO_3_^−^, Br^−^ and F^−^) using high-surface area carbide derived carbon (CDC). The existence of competing ions was reported to lower adsorption capacity of CDC from 6.25 to 4.82 mg/g which accounted for more than 22% reduction. Moreover, SO_4_^2−^ ions was reported to show a greater effect on the adsorption of PO_4_^3−^ in comparison with other anions (NO_3_^−^, Br^−^ and F^−^). Similar findings that monovalent ions are weaker adsorbed at BC compared with multivalent ions were also reported in other studies^22,83–86^.

### Regeneration of BC adsorbent

The regeneration of biochar was studied by immersion of BC in acidic (HCl) and basic (NaOH) solutions that has a 0.1 M concentration. The adsorption capacities of BC after the regeneration with 0.1 M HCl as well as 0.1 M NaOH were 1.56 and 2.85, respectively. The regeneration with NaOH was found to outperform the regeneration with HCl by about 45%. Moreover, the use of 0.1 M NaOH was found to recover about 57% of the PO_4_^3−^ compared with 31% recovery when 0.1 M HCl was used. The regeneration values in the present work was found to correlate with the work reported in literature. For instance, Jiang et al.^87^ regenerated the BC modified with Mg with 3 M NaOH solution. They reported the regeneration of modified BC to account for 55–60%. It was reported that a desorption of 94% of the PO_4_^3−^ in BC was achieved by not only using 3 M NaOH solution but also applying heat (100 °C) for 24 h. Since the aim of present work is to ultimately add BC with adsorbed PO_4_^3−^ to the soil, high regeneration rates are not so critical in the proposed approach.

The successful BC regeneration using aqueous solutions of NaOH at various concentrations (0.1–2 M) was reported by some studies to outperform the acidic regeneration during treatment of wastewater and achieve a removal efficiency of 95% after 4 regeneration cycles^88–97^. For instance, BC modified with iron and magnesium by Jia et al.^91^ was regenerated with 2 M of NaOH achieved a removal efficiency of 76% after 5 regeneration cycles due to the interaction between NaOH and the co-precipitated adsorbate which was reported to keep the chemical structure of the modified biochar intact. Moreover, regeneration of BC loaded with copper, using 0.1 M NaOH was reported by Pan et al.^89^ to achieve a removal efficiency of 85% after 2 regeneration cycles which was reported to take place due to the rigorous activation of NaOH within the biochar; which gave rise to enhanced pore structure connectivity and lowered deformation of BC pores.

The bioavailability of PO_4_^3−^ was also tested by Jiang et al.^87^ who used BC modified with MgO just like the present work. In their work, Mehlich 3 soil test method was used to determine the percentage of desorbed PO_4_^3−^ out of the adsorbent. They reported a desorption/adsorption percentage range of 83–93% which indicated the high bioavailability of PO_4_^3−^ to plants in soil using ecofriendly fertilizer.

## Conclusions

In the present work, a novel BC adsorbent was prepared from *Acacia* *tortilis* trees pruning waste and tested for removal of PO_4_^3−^ from aqueous solutions. The modified BC was doped with Fe_3_O_4_ and MgO by hydrothermal process. The modification of the BC was confirmed by EDS analysis and X-ray diffraction (XRD) techniques. The modification of BC by Fe_3_O_4_ and MgO was found to show a high removal of PO_4_^3−^ ions over a wide concentration range in synthetic aqueous solutions whereas the unmodified BC showed no significant removal. The effect of incorporation of Fe3O4 and MgO onto BC was investigated by analyzing the surface charge and surface area of BC before and after modification. It was observed that the modified BC had higher surface area as well as surface charge when compared with unmodified BC. The BET surface area of the modified BC was 397 m^2^/g compared with 322 m^2^/g before modification. Similarly, the modification of BC with Fe_3_O_4_ and MgO nanoparticles was observed to increase the point of zero electric charge from pH 3.4 (corresponding to plain BC) to pH 5.3 (corresponding to modified BC) due to the presence of positively charged metals such as Fe and Mg which were reported in literature to maximize the attraction between PO ions and BC. The adsorption process was observed to be fast with about 82% and 97.5% removal achieved within adsorption times of 30 min and 4 h, respectively. This can be attributed to the high surface area after the modification of BC. In the present work, a maximum adsorption capacity of 98.5 mg/g was observed at pH 8.5 and PO_4_^3−^ concentration of 500 ppm. The investigation of the best adsorption isotherm models revealed that the adsorption of PO_4_^3−^ ions onto BC can be denoted by Langmuir isotherm model. After confirming the potential of recovering PO_4_^3−^ from aqueous solutions using modified BC, future work will focus on testing the PO_4_^3−^ removal from real treated sewage effluents, adding exhausted BC to the soil and investigating its effect on the plant growth. These investigations will allow the confirmation of the potential use of BC to recover PO_4_^3−^ from wastewater and recycle it back to the soil to make the use of PO_4_^3−^ more sustainable.

### Supplementary Information

Supplementary Figures.

## Data Availability

All data relevant to the study are included in the article or uploaded as supplementary information. In addition, the datasets used and/or analyzed during the current study are available from the corresponding author on reasonable request.
