# Supplementary material for: Adsorptive removal of phosphate from water with biochar from acacia tree modified with iron and magnesium oxides
Source: Sci Rep. 2024 Jul 29;14:17414. doi: 10.1038/s41598-024-66965-3 (PMC11286779; doi:10.1038/s41598-024-66965-3)
Supplement: Supplementary file 1 — Supplementary Figures. [file 41598_2024_66965_MOESM1_ESM.docx]

**Supplementary information**

Figure S1. The deconvolution of C1s and O1s of the sample1.

The deconvolution of C1s and O1s were given in **Fig. S1**. The C1s shows the typical biomass related signal with the C-C, C-O and C=O locating at 284.8eV, 286.4eV and 287.8eV, together with a plasma satellite at 290eV. The doublet at 292eV was assigned to the K2p residual signal. The O1s shows the organic C-O and C=O bonds at 531.4eV and 533.3eV.

Figure S2. The deconvolution of Fe2p, Mg1s, C1s and O1s of Sample2, respectively.

The fittings of Sample 2 are given for the Fe2p, O1s, C1s, and Mg1s in **Fig. S2**. The Fe2p gives the FeII and FeIII components, with the Fe3/2 at 710.3 eV and 712.5 eV, respectively.The Mg1s consists of the Mg-C at 1302 eV and the Mg-O at 1303 eV.The C1s shows the strong signal from metal carbide and the rest of the signal from the biomass material. The corresponding O1s shows the Metal oxidation (Fe-O and Mg-O) at the lower binding energy side and the carbon-related signal at 531.4 eV.A similar signal was observed for Sample 3 in **Fig. S3**. The Mg1s gives a binding energy shift to the higher side due to the newly appearing Mg-OH signal at 1303.6 eV.

Figure S3. Deconvolution of sample 3 with Fe 2p, Mg 1s, C 1s and O 1s.
